# Retrospective Study Shows That Serum Levels of Chemokine CXCL10 and Cytokine GDF15 Support a Diagnosis of Sporadic Inclusion Body Myositis and Immune-Mediated Necrotizing Myopathy

**DOI:** 10.3390/brainsci13101369

**Published:** 2023-09-25

**Authors:** Boel De Paepe, Ken R. Bracke, Jan L. De Bleecker

**Affiliations:** 1 Department of Neurology, Ghent University Hospital, B-9000 Ghent, Belgium; jan.debleecker@ugent.be; 2 Neuromuscular Reference Center, Ghent University Hospital, B-9000 Ghent, Belgium; 3 Department of Respiratory Medicine, Ghent University Hospital, B-9000 Ghent, Belgium; ken.bracke@ugent.be

**Keywords:** biomarker, C-X-C motif chemokine ligand 10, growth differentiation factor 15, idiopathic inflammatory myopathy, immune-mediated necrotizing myopathy, myositis, sporadic inclusion body myositis

## Abstract

The implementation of novel blood-based biomarkers is desired to reduce the diagnostic delay and burden for myositis patients. In this retrospective study, the potential of C-X-C motif chemokine ligand 10 (CXCL10) and growth differentiation factor 15 (GDF15) was explored in an established patient cohort diagnosed with immune-mediated necrotizing myopathy (IMNM; n = 21), sporadic inclusion body myositis (IBM; n = 18), overlap myositis (OM; n = 3), dermatomyositis (DM; n = 2), and anti-synthetase syndrome (ASS; n = 1), comparing these results with healthy controls (n = 10) and patients with a hereditary neuromuscular disorder (n = 14). CXCL10 and GDF15 were quantified in sera with enzyme-linked immunosorbent assays and immunolocalized in skeletal muscle tissue. In myositis patients, serum CXCL10 levels were significantly increased 9.6-fold compared to healthy controls and 4.2-fold compared to disease controls. Mean levels of CXCL10 were 929 ± 658 pg/mL of serum in IBM and 425 ± 324 pg/mL of serum in IMNM. With the threshold set to 180 pg/mL of CXCL10, myositis patients could be differentiated from healthy and disease controls with a sensitivity of 0.80 and a specificity of 0.71. Incorporating a threshold of 300 pg/mL for GDF15 reduced false negatives to two IMNM patients only. Subsets of muscle-infiltrating immune cells expressed CXCL10, and serum levels correlated with muscle inflammation grade. We propose adding circulating CXCL10 and GDF15 to the blood-based diagnostic toolkit for myositis as a valuable patient-friendly approach.

## 1. Introduction

The idiopathic inflammatory myopathies (IIMs) represent a heterogeneous group of distinct autoimmune conditions jointly termed myositis. Subclassification of patients is a necessary effort to develop appropriate disease management and for disease prognosis. Methodologies have been developed for accurate classification, yet they continue to evolve, and debate persists over definitions and validation of diagnostic criteria. Since the subgroups of polymyositis (PM) and dermatomyositis (DM) were first described based upon clinical and myopathological criteria [1,2], a deepened understanding of IIM pathophysiology and heterogeneity has led to further inclusion of in-depth diagnostic imaging and laboratory testing. Autoantibody profiles and muscle magnetic resonance imaging (MRI) have been incorporated successfully into the diagnostic arsenal [3]. The distinct subgroup of sporadic inclusion body myositis (IBM) was recognized, characterized by specific clinical features and the presence of endomysial auto-aggressive inflammation and muscle fiber vacuoles and amyloid deposits [4], as well as the frequent presence of anti-cytosolic 5′–nucleotidase 1A (CN1A) autoantibodies [5]. The subgroup of immune-mediated necrotizing myopathy (IMNM) has also been recognized and is characterized by muscle necrosis predominating over inflammation in the diagnostic biopsies [6], and an association with anti-signal recognition particle (SRP) or anti-3-hydroxy-3-methylglutaryl-coA reductase (HMGCR) autoantibodies in some of the patients [7]. Autoantibodies directed against aminoacyl tRNA synthetases reveal myositis as part of the antisynthetase syndrome (ASS), a subgroup of patients who frequently suffer from interstitial lung disease (ILD) [8]. Myositis may also co-occur with other connective tissue diseases, termed overlap myositis (OM).

A conclusive diagnosis of IIMs may require specialized and elaborate clinical, genetic, histological and biochemical evaluation, and for many patients means undergoing a diagnostic muscle biopsy as a necessary yet invasive and time-consuming effort for which standardized diagnostic procedures have been proposed [9]. Further implementation of blood-based disease biomarkers therefore represents a convenient alternative approach with the potential to further reduce the need for diagnostic muscle biopsies in the myositis patient population. This is a very plausible approach, as a blood sample is routinely taken from patients for the measurement of skeletal muscle markers (including the inevitable creatine kinase) and autoantibody typing, the latter already being an established part of the diagnostic process. This study focuses on two stress-related proteins and their biomarker potential for identifying and subtyping the IIMs. C-X-C chemokine ligand 10 (CXCL10), also known as interferon γ-induced protein 10 (IP-10), is a chemokine with a pathogenic role in autoimmune diseases that features among the main myokines involved in the pathogenesis and progression of myositis [10]. Damaged muscle expresses higher levels of CXCL10, yet the chemokine is dispensable for effective muscle regeneration [11]. A strong association of CXCL10 with IIMs has been known for two decades, with documented expression in skeletal muscle [12,13,14] and elevation of circulating levels in the blood [15,16,17,18]. Growth differentiation factor 15 (GDF15) is a transforming growth factor β superfamily cytokine implicated in age-related disorders, inflammation and cognitive decline [19]. Elevated GDF15 was only recently described in IIMs [20,21], with GDF15 levels associated with an increased risk of myocardial injury [22]. 

In this study, we explore the potential of CXCL10 and GDF15 evaluation in patient sera for diagnosing and subdividing the IIMs. 

## 2. Materials and Methods

### 2.1. Subjects and Materials

This retrospective study included sera and muscle biopsies from an established cohort of 45 adult IIM patients with confirmed clinical and myopathological diagnosis of IMNM (n = 21), IBM (n = 18), OM (n = 3), DM (n = 2) and ASS (n = 1) (Table 1). Serum samples were collected between 2011 and 2022, and were stored at −80° until use. Comparative tests of individual samples showed no significant decay of CXCL10 and GDF15 over this time period. 

Control materials were commercially obtained samples from healthy subjects (Zenbio, Durham, NC, USA) and sera from patients with hereditary muscle disease that were diagnosed in our hospital (Supplementary Table S1). Sampling adhered to ethical and privacy regulations.

### 2.2. Quantification of Serum CXCL10 and GDF15 Levels

Enzyme-linked immunosorbent assays were performed with human GDF15 (DGD150) and CXCL10 (DIP100) Quantikine ELISA kits from R&D Systems (Bio-Techne, Abingdon, UK) according to the manufacturer’s specifications. Based upon preliminary experiments, optimal dilutions were determined (1/10 and 1/20 for control, 1/10 and 1/50 for patient sera). Sera were loaded onto 96-well plates in duplicate. Values were calculated as the mean of duplicates and the two dilutions tested, and were reported as mean ± SD. The Shapiro–Wilk test determined that variables were not normally distributed, and hence the Kruskal–Wallis test by rank for multiple groups of independent values was used, comparing values pairwise between groups. Asymptotic significance values in 2-sided tests were adjusted by means of Bonferroni correction for multiple tests, with mean differences considered significant from the 0.05 level. Bivariate Pearson’s correlation tests were performed to evaluate correlations between variables. Receiver operating characteristic (ROC) analysis was used to compare diagnostic performances, and graphic representation with area under the curve (AUC) measured separability. All analyses were performed with SPSS software version 28 (IBM, New York, NY, USA).

### 2.3. Immunofluorescence, Immunohistochemistry and Histochemistry

Immunostaining was performed on 6 µm frozen muscle sections, first treated with blocking solution containing 5% donkey serum, 10% heat-inactivated human serum and 2% bovine serum albumin in phosphate-buffered saline. Immunofluorescent immunolocalization of GDF15 was carried out with 4 µg/mL mouse monoclonal IgG_2a_ anti-GDF15 (clone H-2; Santa Cruz Biotechnology, Santa Cruz, CA, USA), combined with 0.7 µg/mL rabbit polyclonal anti-CD68 (H-255; Agilent, Santa Clara, CA, USA) or 1 µg/mL rabbit polyclonal anti-CD56 (Fisher Scientific, Waltham, MA, USA) or 1.25 µg/mL rabbit polyclonal anti-LC3B (ab48394, Abcam, Cambridge, UK), and incubated for 2 h at room temperature. Secondary antibodies were labeled with CY3 (Jackson ImmunoResearch Laboratories, West Grove, PA, USA) and AlexaFluor488 (Invitrogen, Carlsbad, CA, USA) and slides were mounted with Fluoromount (Southern Biotech, Birmingham, AL, USA). Serial sections were immunostained with mouse monoclonal IgG_2a_ anti-CXCL10 (4D5; Biorad Laboratories, Temse, Belgium), 4 µg/mL mouse monoclonal IgG_1_ anti-CD68 (KP1, Abcam, Trumpington Cambridge, UK), and 1.3 µg/mL mouse monoclonal IgG_1_ anti-SQSTM1 (BD Biosciences, San Jose, CA, USA) for 1 h (or 2 h for anti-CXCL10) at room temperature. Sections were stained with Envision anti-mouse and DAB substrate (Agilent) according to the manufacturer’s specifications, and mounted with aquatex (Merck Life Science, Hoeilaart, Belgium). Muscle tissues were imaged and recorded with a light/fluorescence microscope (Zeiss, Goettingen, Germany) and analyzed with CellF software (Olympus, Antwerp, Belgium). In a selection of patient biopsies, muscle histology and inflammation were evaluated in hematoxylin and eosin (H&E)-stained sections using standard histological procedures, and scored as absent (0), intermediate (1) to severe (2) by an experienced myopathologist.

## 3. Results

### 3.1. Increased CXCL10 and GDF15 Levels in IIM Sera

**Figure 1:**
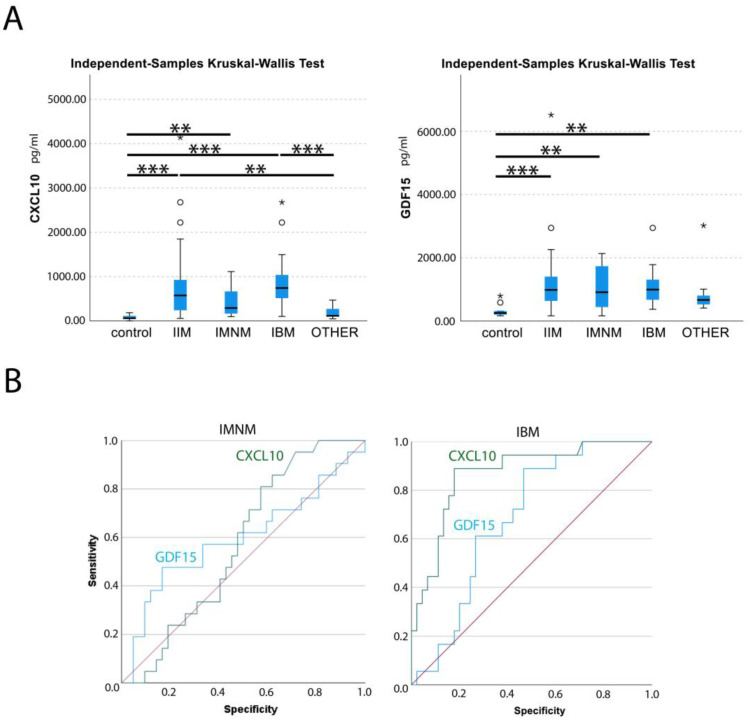
Statistical analysis of circulating levels of CXCL10 and GDF15 in myositis patients (**A**) Box plot of circulating levels of CXCL10 and GDF15 are given in pg per mL of serum in controls, idiopathic inflammatory myopathy (IIM), immune-mediated necrotizing myopathy (IMNM), sporadic inclusion body myositis (IBM) and patients with different hereditary muscle disorders (OTHER). Outliers (circles) and extreme values (stars) have been indicated. Kruskal–Wallis test by rank for multiple groups of independent values, with Bonferroni correction for multiple tests determined significant differences: *p* < 0.05 *, *p* < 0.01 **, *p* < 0.001 ***. (**B**) ROC analysis for CXCL10 (green) and GDF15 (blue) serum levels in IMNM and IBM patients, with reference line (red), are shown. Graphics were generated with SPSS software.

In individual patients and controls, levels of CXCL10 and GDF15 were determined in the same serum sample (Supplementary Table S2). Statistical analysis was performed with Kruskal–Wallis one-way analysis of variance with Bonferroni correction for multiple tests (Figure 1A), and ROC analysis compared diagnostic performance (Figure 1B).

The mean circulating levels of CXCL10 were 79 ± 53 pg/mL for healthy controls, 180 ± 123 pg/mL for patients with hereditary muscle disorders and 755 ± 783 pg/mL for IIM patients. In IMNM, values were increased 5.4-fold compared to healthy controls, and 2.4-fold compared to disease controls. In IBM, CXCL10 levels were increased a further 11.7-fold compared to healthy controls and 5.2-fold compared to disease controls. Only weak correlations between CXCL10 serum levels and clinical characteristics could be observed (Supplementary Table S3), yet at times in different directions. A weak negative correlation with BMI was observed in IIMs, while in hereditary muscle disorders, a weak positive correlation was found (r = 0.22). A weak positive correlation with cardiac disease was observed in hereditary muscle disorders (r = 0.20), while a weak negative correlation was present in IIMs and IBM (r = −0.24).

The mean circulating GDF15 levels were 326 ± 204 pg/mL for healthy controls, 831 ± 656 pg/mL for patients with hereditary muscle disorders and 1201 ± 1017 pg/mL for IIM patients. Values were comparably increased in subgroups by 3.2-fold (IMNM) and 3.4-fold (IBM) compared to healthy controls, and 1.3-fold compared to disease controls. GDF15 levels were moderately correlated with age at sampling in IMNM and OTHER (r = 0.53) (Supplementary Table S3). When the IIMs were combined, the correlation with age was only weak (r = 0.26). In IMNM, a weak correlation of GDF15 with blood CK values was noted (r = 0.22). A weak correlation was observed with cardiac disease in the IIMs (r = 0.27) and the subgroup of IBM (r = 0.36). 

Levels of CXCL10 and GDF15 were not correlated in any of the sera from all diagnostic groups. ROC analysis found AUCs for CXCL10 were 0.573 for IMNM and 0.870 for IBM, and 0.879 for the whole group of IIMs. With the threshold set to 180 pg/mL of CXCL10, myositis patients could be differentiated from healthy and disease controls with a sensitivity of 0.80 and a specificity of 0.71. For GDF15, AUC were 0.596 for IMNM and 0.688 for IBM, and 0.772 in combined IIMs. 

### 3.2. Localization of CXCL10 to Muscle Fibers and Actively Invading Inflammatory Cells

**Figure 2:**
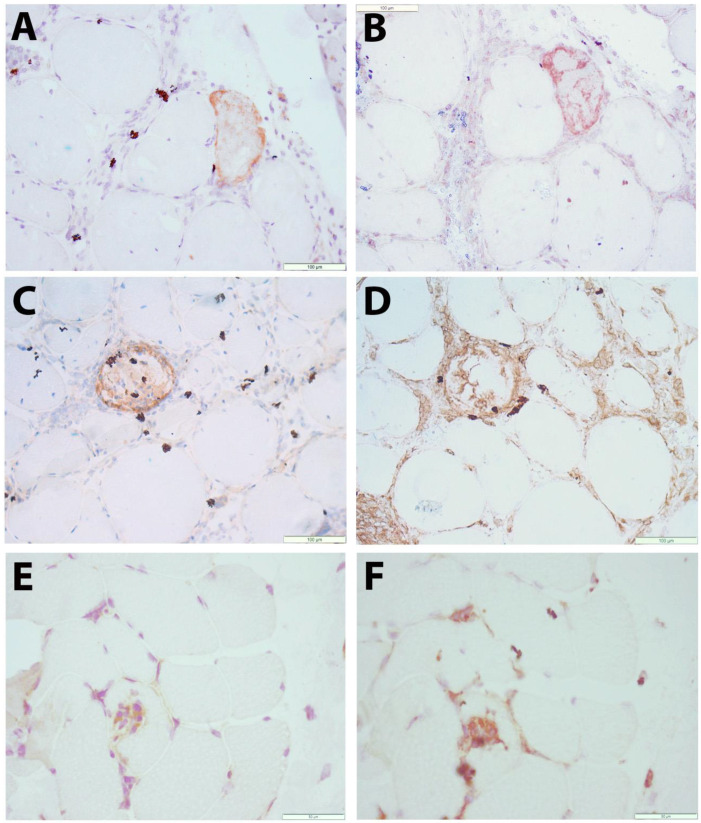
Immunolocalization of CXCL10 in skeletal muscle tissue (**A**–**D**) Immune-mediated necrotizing myopathy (IMNM21): a muscle fiber stains for CXCL10 (brown in **A**). In a sequential section, this fiber is shown to be SQSTM1-positive (brown in **B**). CXCL10 staining (brown in **C**) is observed in a necrotic muscle fiber. From a sequential section stained with macrophage marker CD68 (brown in **D**), it can be observed that macrophages are mostly CXCL10-negative. (**E**,**F**) Sporadic inclusion body myositis (IBM15): immune cells actively invading a nonnecrotic muscle fiber are partly CXCL10-positive (brown in **E**). A sequential section stained with macrophage marker CD68 (brown in **F**) shows partial colocalization. Scale bars = 100 µm (**A**–**D**), 50 µm (**E**,**F**).

To allow the evaluation of CXCL10 expression alongside pathological changes to the muscle tissue, immunohistochemical staining was performed in sequential muscle sections. Muscle biopsies with normal histology were largely CXCL10-negative. In contrast, subsets of small muscle fibers in IIM tissues displayed a granular staining pattern in necrotic muscle fibers and in SQSTM1-positive muscle fibers (Figure 2A–D).

**Figure 3:**
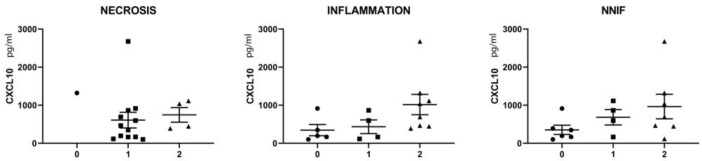
Relationship between serum CXCL10 levels and the scoring of myopathological changes in skeletal muscle tissues of a selection of IMNM (n = 9) and IBM (n = 8) patients. CXCL10 values are given in pg/mL of serum. Muscle fiber necrosis, buildup of intramuscular inflammation and the presence of non-necrotic invaded muscle fibers (NNIF) were scored in individual patients as absent (0, indicated by circles) intermediate (1, indicated by squares) or severe (2, indicated by triangles). For detailed scoring results, consult Supplementary Table S4.

The pattern of myopathological changes differed between IMNM and IBM patients (Supplementary Table S4). IMNM was associated with muscle fiber necrosis and less severe inflammatory damage, while IBM was strongly associated with endomysial buildup of inflammation and active invasion of non-necrotic muscle fibers by auto-aggressive immune cells. In IBM tissues, a subset of inflammatory cells were CXCL10-positive, notably immune cells invading non-necrotic muscle fibers, of which most were CD68-positive (Figure 2E,F). The severity of inflammatory changes in individual IIM patients tended to be associated with circulating levels of CXCL10 (Figure 3), though no significance was shown in this smaller patient sample.

### 3.3. Co-Localization of GDF15 with Markers of Autophagy and Regeneration in Muscle Fibers

**Figure 4:**
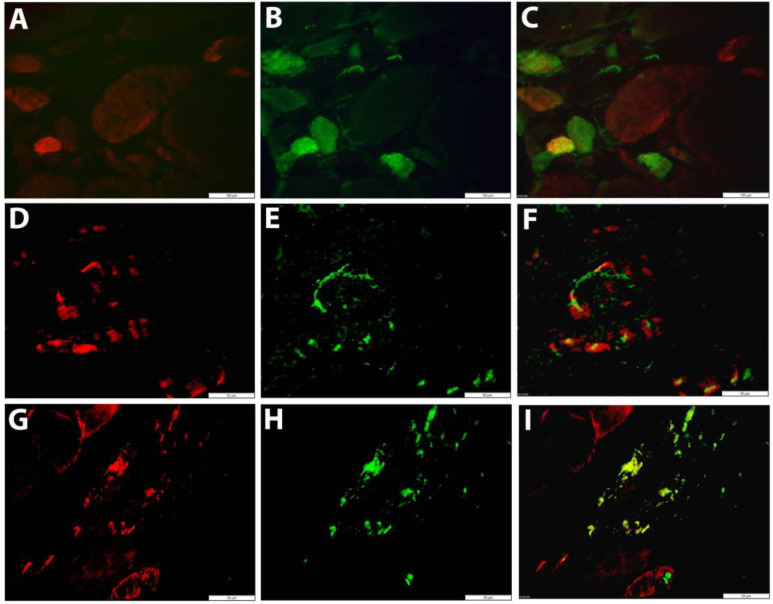
Fluorescent immunolocalization of GDF15 in skeletal muscle tissue (**A**–**C**) Immune-mediated necrotizing myopathy (IMNM17): a small fiber stains strongly for GDF15 (red in **A**), which is CD56-positive (green in **B**). The double stain (**C**) shows lower or absent expression in other regenerating muscle fibers. (**D**–**I**) Sporadic inclusion body myositis (IBM01): granular GDF15 staining is observed in muscle fibers (red in **D**,**G**), co-localizing with LC3B (green in **E**) and SQSTM1 (green in **H**). Double staining shows GDF15 and SQSTM1 immunostaining overlaps in protein aggregates (yellow in **I**). Scale bars = 100 µm (**A**–**C**), 50 µm (**D**–**I**).

The low constitutive sarcoplasmic GDF15 staining observed in healthy controls was notably increased in IIM muscle biopsies, mostly in small regenerating muscle fibers (Figure 4A–C). A granular staining pattern was observed in other subsets of muscle fibers, co-localizing with autophagic markers (Figure 4D–I). The vast majority of inflammatory cells were GDF15-negative.

## 4. Discussion

Subtyping of IIMs is a necessary effort to design treatment strategies suited to the individual patient. While subgroups of patients react well to standard immunosuppressive therapies, others might require alternative immunomodulatory strategies. In IMNM, the autoantibody status aids as an indicator whether the response to different treatment regimens would be favorable [23]. IBM is largely unresponsive to current immunomodulatory treatment. In addition to subclassification, it is imperative to differentiate IIMs from muscular dystrophies to avoid inappropriate treatment with glucocorticoids in the latter. Circulating biomarkers have been in use for diagnosing myositis for decades, with blood samples routinely taken to evaluate CK and other muscle enzymes. However, this strategy has certainly not yet been developed to its full potential. In this respect, implementing the analysis of the expression of key pathogenic factors in patient sera is an attractive prospect. A good choice would be to analyze myokines, i.e., cytokines and other proteins produced and released by muscle cells which enable the skeletal muscle tissue to communicate with the body’s other organs, as indicators of muscle dysfunction [24]. 

Circulating CXCL10 has already been described as a reliable and sensitive biomarker for IIM subgroups. In a study of 125 patients diagnosed with juvenile DM, serum CXCL10 levels displayed 0.87 sensitivity and 1.00 specificity for active disease [17]. Our current study confirmed the association with IIMs and indicated higher levels in the subgroup of IBM in comparison to IMNM. The number of included patients with other subgroups of IIMs was too low to allow other comparisons. Additional studies would be of interest, especially in DM and ASS patients subtyped based upon autoantibody profiles. In the skeletal muscle tissue of IBM and IMNM patients, we observed CXCL10 staining in necrotic fibers, yet this needs to be interpreted with caution, as unspecific staining is often observed in necrosis. Though CXCL10 is present in muscle fibers and a subset of inflammatory cells, it remains enigmatic if the muscle tissue is an important source of the chemokine, or if intramuscular inflammation is more a consequence of systemic CXCL10 expression. CXCL10 elevation as an indicator of muscle disease severity goes beyond the IIMs. In systemic sclerosis, serum CXCL10 levels strongly correlate with clinical severity of muscle involvement and with CK serum concentration, suggesting a potential mechanistic involvement in muscle damage [25]. 

No single diagnostic feature can differentiate IIMs, let alone reliably subtype the different subgroups. A threshold of 180 pg/mL of CXCL10 differentiates myositis patients from healthy and disease controls with a sensitivity of 0.80 and a specificity of 0.71. Importantly, we showed that CXCL10 levels aid in differentiating IIMs from hereditary muscle disorders, with the latter often displaying secondary inflammatory changes that can be confused with myositis. We found CXCL10 levels in hereditary muscle disorders to be no different from in healthy controls; however, another study reported CXCL10 to be significantly elevated in serum and muscle samples of DMD patients, relative to age-matched healthy controls [26]. We speculate that adding CXCL10 to the diagnostic toolkit might be useful, but might not be able to boost diagnostic performance sufficiently. We propose that circulating CXCL10 could, however, be part of a bigger strategy for evaluating effective combinations of biomarkers. In this respect, our results appoint GDF15 consideration as an additional, more general marker for muscle disorders [27]. GDF15 is currently explored as a biomarker in many disorders including cardiovascular disease [28], cancer [29] and mitochondrial myopathy [30].

When considering novel circulating biomarkers, it is imperative to determine normal value variations in the healthy population. Many factors may influence serum levels, including gender, age and physical activity. It is known that the complex mixture of myokines secreted into the bloodstream varies during muscle contraction [31]. In this respect, GDF15 and CXCL10 seem to be somewhat opposite poles. While GDF15 gene expression is induced in muscle tissues of mice when exercised [32] and in response to oxidative stress [33], in contrast, treadmill running significantly reduced CXCL10 gene expression in mice soleus muscle [34]. Either way, circulating GDF15 and CXCL10 both appear to be regulated by physical activity. Nonetheless, CXCL10 levels have been observed to remain stable among healthy controls [26], while GDF15 values appear more prone to changes in humans. In pregnant women, blood levels rise rapidly and stay high during the whole pregnancy [35]. In addition, GDF15 levels are associated with aging and tend to increase across the lifespan. Elevated GDF15 has been observed to correlate with reduced muscle strength and extremity function in older patients with cardiometabolic disease [36] and to be associated with lower muscle mass in men specifically [37], the latter being a further indication of sex differences. A limitation of our study is the age variation between diagnostic groups, with average ages of healthy controls (34 ± 12) and patients with hereditary muscle disorders (45 ± 13) being substantially lower than those of IMNM (65 ± 9) and IBM (71 ± 7) patients. In IMNM patients and the group of patients with hereditary muscle disorders, we found a moderate correlation of GDF15 serum levels with age at sampling. An effort to determine values that can be used as reference ranges was published recently [38], with the most notable increases in the aging population associated with heart disease and diabetes. Another characteristic described as being associated with elevated circulating GDF15 levels is obesity [39,40]. In our IIM cohort, 51% of patients were overweight, of which 13.3% were obese (defined by a BMI over 30), yet we did not find a correlation between BMI and serum GDF15 levels.

We propose that our study may contribute to patient-friendly diagnostic innovation. Further minimization and multiplex immunoassays could allow the expansion and analysis of combinations of biomarkers. In this respect, blood spot analysis could be put forward as a convenient approach, as sampling can be performed by nontrained persons and the material can be stored and transported at ambient temperatures. Studies evaluating spotted TNFα confirmed that this methodology can detect cytokine concentrations commonly observed in patient samples, which range from 5 to 27 pg/mL [41]. For CXCL10, a high correlation of blood spot analysis with serum levels (r = 0.96) has already been described [17]. Another innovation could be to attempt the least invasive sample collection available, which involves analyzing a urine sample. The urine proteome as a possible source of biomarkers has been explored for the juvenile form of DM [42]. In chronic kidney disease, urine GDF15 levels have already been shown predictors of mortality with an AUC of 0.95 [43]. 

In addition to the diagnostic purposes of biomarker studies, serum biomarkers can be useful as follow-up therapeutic markers in clinical trials, with the comparison of levels pre- and post-treatment as exploratory outcome measures in individual patients. Additionally, biomarker studies advance our understanding of pathogenic changes in IIM patients and may identify novel therapeutic targets. Myokines involved in the immunopathological processes triggered by the immune system, aggravating or ameliorating inflammatory muscle disease, may become important therapeutic targets in their own right, representing an appropriate personalized therapeutic strategy [10]. Myokines evolving from biomarkers to therapeutic targets have been proposed for cancer cachexia [44]. 

## 5. Conclusions

Our study found significant elevation of serum CXCL10 and GDF15 levels in myositis patients. The skeletal muscle tissue is one of the possible sources, with localization to subsets of affected muscle fibers and inflammatory cells. CXCL10 expression was notably high in immune cells invading non-necrotic muscle fibers and appeared correlated with muscle tissue inflammation grade. We propose that circulating CXCL10 and GDF15 levels could be of aid to diagnose myositis. If our findings were to be confirmed, GDF15 could be developed into a more general biomarker for muscle disease and CXCL10 levels as an indicator for IIM subtypes characterized by severe muscle inflammation and the active invasion of muscle fibers by auto-aggressive immune cells. Further implementation of circulating biomarkers might reduce the need for taking a diagnostic muscle biopsy further, at least in part of the patients.

## Figures and Tables

**Table 1:** Patient clinical data.

Diagnosis	ID	Gender	Age	BMI	CK	Autoantibodies	Medication	Associated Disease/Comorbidities
IMNM	01	F	67	33	1417	HMGCR+	GC IVIG	Myocardial infarction, Hashimoto’s thyroiditis, diabetes
	02	F	77	25	2614	HMGCR+		
	03	F	60	24	6923	HMGCR+		Cancer
	04	M	61	27	851	HMGCR+	STAT	Cerebellar ataxia, diabetes, obstructive sleep apnea
	05	F	76	30	7000	HMGCR+	STAT GC	Sjogren’s syndrome, diabetes
	06	F	74	22	10,899	HMGCR+	STAT	Diabetes
	07	M	73	31	7855	HMGCR+	STAT	
	08	F	70	25	9356	HMGCR+	STAT	Pneumocystis pneumonia, ischemic heart disease, diabetes
	09	F	72	20	5572	HMGCR+	STAT	
	10	M	68	26	1889	HMGCR+	STAT	Coronary heart disease
	11	M	73	>25	4876	HMGCR+	STAT	Coronary heart disease, diabetes
	12	F	60	22	5749	HMGCR+	GC	
	13	F	56	23	6144	SRP+	GC IVIG TNF	
	14	F	65	20	6168	SRP+	STAT	
	15	M	67	23	609	SAE1+	STAT GC	Diabetes
	16	F	68	31	100	Ro52+	GC	Sudeck dystrophy, diabetes, diverticulitis
	17	F	53	22	233	PM/Scl75+		
	18	F	74	21	3000	ND	GC	Hypothyroidism, heart failure
	19	M	46	26	10,264	ND	GC	RA
	20	M	57	29	400	ND	GC	RA, atherosclerosis
	21	F	53	31	150	ND	GC	
IBM	01	M	73	21	170	cN1A+	GC	
	02	F	68	19	262	cN1A+		
	03	M	72	22	128	cN1A+	GC	
	04	M	76	25	186	cN1A+		
	05	M	62	25	513	cN1A+		
	06	F	61	20	717	cN1A-		
	07	F	75	21	290	cN1A-		RA
	08	F	82	24	160	cN1A-		
	09	F	70	25	658	cN1A-		Cancer
	10	M	76	>25	399	cN1A-	GC β-BL	Pericarditis
	11	M	72	25	68	cN1A-	STAT GC	Hypercholestrolemia
	12	M	66	24	579	cN1A-	IVIG	Psoriasis, diabetes, atherosclerosis
	13	F	64	31	134	cN1A-	STAT	Hashimoto’s thyroiditis
	14	M	70	22	118	ND	GC	RA, COPD
	15	M	73	<25	303	ND		
	16	M	66	<25	626	ND		Diabetes, hypercholesterolemia
	17	M	61	26	356	ND		
	18	M	84	>25	180	ND		Myocardial infarction, Hashimoto’s thyroiditis
OM	01	F	42	23	542		GC	Sjogren’s syndrome
	02	M	56	24	462	cN1A-		Spondyloarthritis, coronary heart disease
	03	M	70	27	308	SSA+ Ro52+		RA, cancer, diabetes
DM	01	F	57	<25	2139	Mi2+	GC	
	02	M	44	ND	1616	ND		
ASS	01	F	61	23	3046	Ro52+ Jo1+		ILD

Abbreviations: anti-synthetase syndrome (ASS), β-blockers (β-BL), body mass index (BMI), chronic obstructive pulmonary disease (COPD), creatine kinase (CK), cytosolic 5′-nucleotidase 1A (cN-1A), dermatomyositis (DM), female (F), glucocorticoids (GC), hydroxy-3-methylglutaryl-coenzyme A reductase (HMGCR), immune-mediated necrotizing myopathy (IMNM), sporadic inclusion body myositis (IBM), interstitial lung disease (ILD), intravenous immunoglobulin (IVIG), male (M), not determined (ND), overlap myositis (OM), rheumatoid arthritis (RA), small ubiquitin-like modifier 1 activating enzyme (SAE), signal recognition particle (SRP), Sjogren’s-syndrome-related antigen A (SSA), statins (STAT), TNF inhibitors (TNF). Age is given in years. Only medication taken prior to sampling is listed.

## Data Availability

The data presented in this study are available on request from the corresponding author.

## Supplementary Materials

The following supporting information can be downloaded at: https://www.mdpi.com/article/10.3390/brainsci13101369/s1, Table S1: Healthy and disease controls; Table S2: Quantification of circulating CXCL10 and GDF15 in human sera using enzyme-linked immunosorbent assays; Table S3: Pearson’s correlation coefficients between variables; Table S4: Scoring myopathological changes in muscle biopsies from a selection of patients.

## References

[1] Bohan A., Peter J.B. (1975). Polymyositis and dermatomyositis (first of two parts). N. Engl. J. Med..

[2] Bohan A., Peter J.B. (1975). Polymyositis and dermatomyositis (second of two parts). N. Engl. J. Med..

[3] Targoff I.N., Miller F.W., Medsger T.A., Oddis C.V. (1997). Classification criteria for the idiopathic inflammatory myopathies. Curr. Opin. Rheumatol..

[4] Griggs R.C., Askanas V., DiMauro S., Engel A., Karpati G., Mendell J.R., Rowland L.P. (1995). Inclusion body myositis and myopathies. Ann. Neurol..

[5] Amlani A., Choi M.Y., Tarnopolsky M., Brady L., Clarke A.E., Garcia-De La Torre I., Mahler M., Schmeling H., Barber C.E., Jung M. (2019). Anti-NT5c1A Autoantibodies as Biomarkers in Inclusion Body Myositis. Front. Immunol..

[6] Hoogendijk J.E., Amato A.A., Lecky B.R., Choy E.H., Lundberg I.E., Rose M.R., Vencovsky J., de Visser M., Hughes R.A. (2004). 119th ENMC international workshop: Trial design in adult idiopathic inflammatory myopathies, with the exception of inclusion body myositis, 10–12 October 2003, Naarden, The Netherlands. Neuromuscul. Disord..

[7] Liu M., Lin Y., Qiao L., Chen J., Shi Q. (2023). Characteristics of cardiac involvement in immune-mediated necrotizing myopathy. Front. Immunol..

[8] Wells M., Alawi S., Thin K.Y.M., Gunawardena H., Brown A.R., Edey A., Pauling J.D., Barratt S.L., Adamali H.I. (2022). A multidisciplinary approach to the diagnosis of antisynthetase syndrome. Front. Med..

[9] De Bleecker J.L., De Paepe B., Aronica E., de Visser M., Amato A., Aronica E., Benveniste O., De Bleecker J., de Boer O., De Paepe B. (2015). 205th ENMC International Workshop: Pathology diagnosis of idiopathic inflammatory myopathies Part II 28–30 March 2014, Naarden, The Netherlands. Neuromuscul. Disord..

[10] Mageriu V., Manole E., Bastian A.E., Staniceanu F. (2020). Role of myokines in myositis pathogenesis and their potential to be new therapeutic targets in idiopathic inflammatory myopathies. J. Immunol. Res..

[11] Deyhle M.R., Hafen P.S., Parmley J., Preece C.N., Robison M., Sorensen J.R., Jackson B., Eggett D.L., Hancock C.R., Hyldahl R.D. (2018). CXCL10 increases in human skeletal muscle following damage but is not necessary for muscle regeneration. Physiol. Rep..

[12] Raju R., Vasconcelos O., Granger R., Dalakas M.C. (2003). Expression of IFN-γ-inducible chemokines in inclusion body myositis. J. Neuroimmunol..

[13] De Paepe B., De Keyzer K., Martin J.J., De Bleecker J.L. (2005). Alpha-chemokine receptors CXCR1–3 and their ligands in idiopathic inflammatory myopathies. Acta Neuropathol..

[14] Limongi F. (2015). The CXCR3 chemokines in inflammatory myopathies. Clin. Ter..

[15] Szodoray P., Philip Alex P., Nicholas Knowlton N., Centola M., Dozmorov I., Csipo I., Nagy A.T., Constantin T., Ponyi A., Nakken B. (2010). Idiopathic inflammatory myopathies, signified by distinctive peripheral cytokines, chemokines and the TNF family members B-cell activating factor and a proliferation inducing ligand. Rheumatology.

[16] Uruha A., Noguchi S., Sato W., Nishimura H., Mitsuhashi S., Yamamura T., Nishino I. (2015). Plasma IP-10 level distinguishes inflammatory myopathy. Neurology.

[17] Wienke J., Bellutti Enders F., Lim J., Mertens J.S., van den Hoogen L.L., Wijngaarde C.A., Yeo J.G., Meyer A., Otten H.G., Fritsch-Stork R.D.E. (2019). Galectin-9 and CXCL10 as Biomarkers for Disease Activity in Juvenile Dermatomyositis: A Longitudinal Cohort Study and Multicohort Validation. Arthritis Rheumatol..

[18] Zhou J., Zhao L., Xiao Y., Xie S., Long Y., Wei Y., Meng Q., Li X., Luo H., Zhu H. (2022). The Expression of Cytokine Profiles and Related Receptors in Idiopathic Inflammatory Myopathies. Front. Pharmacol..

[19] Fuchs T., Trollor J.N., Crawford J., Baune B.T., Samaras K., Campbell L., Breit S.N., Brodaty H., Sachdev P., Smith E. (2014). Macrophage inhibitory cytokine-1 is associated with cognitive impairment and predicts cognitive decline—The Sydney Memory and Aging Study. Neurol. Psychiatr. Brain Res..

[20] De Paepe B., Verhamme F., De Bleecker J.L. (2020). The myokine GDF-15 is a potential biomarker for myositis and associates with the protein aggregates of sporadic inclusion body myositis. Cytokine.

[21] Oikawa Y., Izumi R., Koide M., Hagiwara Y., Kanzaki M., Suzuki N., Kikuchi K., Matsuhashi T., Akiyama Y., Ichijo M. (2020). Mitochondrial dysfunction underlying sporadic inclusion body myositis is ameliorated by the mitochondrial homing drug MA-5. PLoS ONE.

[22] Qiu M., Sun X., Qi X., Liu X., Zhang Y., Zhang N., Lu F., Liu W., Changjing F., Wang Q. (2021). The diagnostic value of GDF-15 for myocardial involvement in idiopathic inflammatory myopathy. Rheumatology.

[23] Weeding E., Tiniakou E. (2021). Therapeutic Management of Immune-Mediated Necrotizing Myositis. Curr. Treat. Options Rheumatol..

[24] Coelho-Junior H.J., Picca A., Calvani R., Uchida M.C., Marzetti E. (2019). If my muscle could talk: Myokines as a biomarker of frailty. Exp. Gerontol..

[25] Corinaldesi C., Ross R.L., Abignano G., Antinozzi C., Marampon F., di Luigi L., Buch M.H., Riccieri V., Lenzi A., Crescioli C. (2021). Muscle Damage in Systemic Sclerosis and CXCL10: The Potential Therapeutic Role of PDE5 Inhibition. Int. J. Mol. Sci..

[26] Ogundele M., Zhang J.S., Goswami M.V., Barbieri M.L., Dang U.J., Novak J.S., Hoffman E.P., Nagaraju K., Hathout Y., CINRG-DNHS Investigators (2021). Validation of Chemokine Biomarkers in Duchenne Muscular Dystrophy. Life.

[27] De Paepe B. (2022). The Cytokine Growth Differentiation Factor-15 and Skeletal Muscle Health: Portrait of an Emerging Widely Applicable Disease Biomarker. Int. J. Mol. Sci..

[28] May B.M., Pimentel M., Zimerman L.I., Rohde L.E. (2021). GDF-15 as a Biomarker in Cardiovascular Disease. Arq. Bras. Cardiol..

[29] Wang Y., Jiang T., Jiang M., Gu S. (2019). Appraising growth differentiation factor 15 as a promising biomarker in digestive system tumors: A meta-analysis. BMC Cancer.

[30] Li Y., Li S., Qiu Y., Zhou M., Chen M., Hu Y., Hong S., Jiang L., Guo Y. (2022). Circulating FGF21 and GDF15 as Biomarkers for Screening, Diagnosis, and Severity Assessment of Primary Mitochondrial Disorders in Children. Front. Pediatr..

[31] Pedersen B., Febbraio M. (2012). Muscles, exercise and obesity: Skeletal muscle as a secretory organ. Nat. Rev. Endocrinol..

[32] Gil C.I., Ost M., Kasch J., Schumann S., Heider S., Klaus S. (2019). Role of GDF15 in active lifestyle induced metabolic adaptations and acute exercise response in mice. Sci. Rep..

[33] Morrow R.M., Picard M., Derbeneva O., Leipzig J., McManus M.J., Gouspillou G., Barbat-Artigas S., Dos Santos C., Hepple R.T., Murdock D.G. (2017). Mitochondrial energy deficiency leads to hyperproliferation of skeletal muscle mitochondria and enhanced insulin sensitivity. Proc. Natl. Acad. Sci. USA.

[34] Ishiuchi Y., Sato H., Tsujimura K., Kawaguchi H., Matsuwaki T., Yamanouchi K., Nishihara M., Nedachi T. (2018). Skeletal muscle cell contraction reduces a novel myokine, chemokine (C-X-C motif) ligand 10 (CXCL10): Potential roles in exercise-regulated angiogenesis. Biosci. Biotechnol. Biochem..

[35] Moore A.G., Brown D.A., Fairlie W.D., Bauskin A.R., Brown P.K., Munier M.L.C., Russell P.K., Salamonsen L.A., Wallace E.M., Breit S.N. (2000). The Transforming Growth Factor-β Superfamily Cytokine Macrophage Inhibitory Cytokine-1 Is Present in High Concentrations in the Serum of Pregnant Women. J. Clin. Endocrinol. Metab..

[36] Oba K., Ishikawa J., Tamura Y., Fujita Y., Ito M., Iizuka A., Fujiwara Y., Kodera R., Toba A., Toyoshima K. (2020). Serum growth differentiation factor 15 level is associated with muscle strength and lower extremity function in older patients with cardiometabolic disease. Geriatr. Gerontol. Int..

[37] Herpich C., Franz K., Ost M., Otten L., Coleman V., Klaus S., Müller-Werdan U., Norman K. (2021). Associations Between Serum GDF15 Concentrations, Muscle Mass, and Strength Show Sex-Specific Differences in Older Hospital Patients. Rejuvenation Res..

[38] Welsh P., Kimenai D.M., Marioni R.E., Hayward C., Campbell A., Porteous D., Mills N.L., O’Rahilly S., Sattar N. (2022). Reference ranges for GDF-15, and risk factors associated with GDF-15, in a large general population cohort. Clin. Chem. Lab. Med..

[39] Vila G., Riedl M., Anderwald C., Resl M., Handisurya A., Clodi M., Prager G., Ludvik B., Krebs M., Luger A. (2011). The relationship between insulin resistance and the cardiovascular biomarker growth differentiation factor-15 in obese patients. Clin. Chem..

[40] Carballo-Casla A., García-Esquinas E., Buño-Soto A., Struijk E.A., López-García E., Rodríguez-Artalejo F., Ortolá R. (2022). Metabolic syndrome and Growth Differentiation Factor 15 in older adults. GeroScience.

[41] Badowski M., Darbouze L., Harris D.T. (2020). Evaluation of biobanked blood spot cards to detect cytokines in blood. Cytotherapy.

[42] Morales M., Alayi T.D., Tawalbeh S.M., Sydenstricker A.V., Spathis R., Kim H., Nagaraju K., Hathout Y., Rider L.G. (2023). Urine proteomics by mass spectrometry identifies proteins involved in key pathogenic pathways in patients with juvenile dermatomyositis. Rheumatology.

[43] Perez-Gomez M.V., Pizarro-Sanchez S., Gracia-Iguacel C., Cano S., Cannata-Ortiz P., Sanchez-Rodriguez J., Sanz A.B., Sanchez-Niño M.D., Ortiz A. (2021). Urinary Growth Differentiation Factor-15 (GDF15) levels as a biomarker of adverse outcomes and biopsy findings in chronic kidney disease. J. Nephrol..

[44] Manole E., Ceafalan L.C., Popescu B.O., Dumitru C., Bastian A.E. (2018). Myokines as Possible Therapeutic Targets in Cancer Cachexia. J. Immunol. Res..

