# Supplementary material for: Retrospective Study Shows That Serum Levels of Chemokine CXCL10 and Cytokine GDF15 Support a Diagnosis of Sporadic Inclusion Body Myositis and Immune-Mediated Necrotizing Myopathy
Source: Brain Sci. 2023 Sep 25;13(10):1369. doi: 10.3390/brainsci13101369 (PMC10605230; doi:10.3390/brainsci13101369)
Supplement: Supplementary file 1 [file brainsci-13-01369-s001.zip › brainsci-2620096-supplementary.pdf]

Table S1: Healthy and disease controls

Diagnosis	ID	Gender	Age	BMI	CK	Muscle disease and causal gene variant
Healthy controls	CONTROL01	F	36	>20<25	ND	none
	CONTROL02	F	22	>20<25	ND	none
	CONTROL03	M	40	>20<25	ND	none
	CONTROL04	F	24	>20<25	ND	none
	CONTROL05	F	23	>20<25	ND	none
	CONTROL06	M	37	>20<25	ND	none
	CONTROL07	F	28	>20<25	ND	none
	CONTROL08	M	41	>20<25	ND	none
	CONTROL09	F	61	>20<25	ND	none
	CONTROL10	F	25	>20<25	ND	none
Hereditary muscle disorders	OTHER01	M	63	24	600	Becker MD *DMD* del exons48-52
	OTHER02	M	42	27	1108	Becker MD *DMD* del exons45-48
	OTHER03	M	29	24	1412	Becker MD *DMD* del exons45-48
	OTHER04	M	44	34	587	Becker MD *DMD* del exons45-48
	OTHER05	M	26	<25	16239	LGMDR12 *ANO5* c.191dupA
	OTHER06	M	44	23	3466	LGMDR9 *FKRP* c.826C>A
	OTHER07	M	52	<20	1968	LGMDR9 *FKRP* c.826C>A
	OTHER08	M	39	26	4855	LGMDR26 *BVES* c.1A>G
	OTHER09	M	32	<20	2250	X-linked Emery Dreiffuss
	OTHER10	F	31	17	366	facioscapulohumeral muscular dystrophy 1
	OTHER11	M	55	28	232	Pompe disease *GAA* c.-32T>G, del exon18
	OTHER12	M	59	25	327	Pompe disease *GAA* c.-32T>G, del exon18
	OTHER13	F	58	21	105	Bethlem myopathy *COL6A3* c.6175G>T
	OTHER14	M	60	32	415	hereditary IBM *VCP* c.374G>A

Age is given in years. Abbreviations: Body mass index (BMI), Creatine kinase (CK), female (F), male (M)

Table S2: Quantification of circulating CXCL10 and GDF15 in human sera using enzyme-linked immuno sorbent assays

Diagnosis	Subgroup	ID	CXCL10*	GDF15*
Healthy controls		CONTROL01	184	169
		CONTROL02	47	170
		CONTROL03	48	317
		CONTROL04	133	272
		CONTROL05	44	206
		CONTROL06	112	235
		CONTROL07	56	290
		CONTROL08	65	587
		CONTROL09	0	795
		CONTROL10	96	215
			**79±53**	**326±204**
IIM	IMNM	IMNM01	165	1403
		IMNM02	291	1121
		IMNM03	948	368
		IMNM04	102	1735
		IMNM05	251	912
		IMNM06	575	1840
		IMNM07	797	1778
		IMNM08	249	1947
		IMNM09	832	2136
		IMNM10	665	1338
		IMNM11	231	1989
		IMNM12	101	207
		IMNM13	388	445
		IMNM14	383	1123
		IMNM15	347	609
		IMNM16	163	689
		IMNM17	194	271
		IMNM18	914	360
		IMNM19	96	167
		IMNM20	114	832
		IMNM21	1113	562
			**425±324**	**1040±661**
	IBM	IBM01	597	640
		IBM02	2221	1784
		IBM03	1496	373
		IBM04	864	675
		IBM05	1320	543
		IBM06	691	1309
		IBM07	621	1106
		IBM08	2677	632
		IBM09	926	1055
		IBM10	240	1144
		IBM11	1037	977
		IBM12	443	1002
		IBM13	463	742

		IBM14	101	790
		IBM15	513	986
		IBM16	1035	1781
		IBM17	793	1389
		IBM18	677	2945
			**929±658**	**1104±603**
	OM	OM01	1783	978
		OM02	361	829
		OM03	55	6516
	DM	DM01	1848	1100
		DM02	172	671
	ASS	ASS01	4139	2260
			**755±783**	**1201±1017**
Hereditary muscle disorders		OTHER01	99	3020
		OTHER02	270	554
		OTHER03	100	438
		OTHER04	119	482
		OTHER05	349	807
		OTHER06	97	413
		OTHER07	197	762
		OTHER08	185	556
		OTHER09	46	631
		OTHER10	293	528
		OTHER11	110	966
		OTHER12	78	764
		OTHER13	102	709
		OTHER14	469	1009
			**180±123**	**831±656**

*Values are given in pg/ml. Abbreviations: anti-synthetase syndrome (ASS), dermatomyositis (DM), sporadic inclusion body myositis (IBM), idiopathic inflammatory myopathies (IIM), immune-medicated necrotizing myopathy (IMNM), overlap myositis (OM).

Table S3: Pearson's correlation coefficients between variables

	IIM		IMNM		IBM		OTHER	
	CXCL10	GDF15	CXCL10	GDF15	CXCL10	GDF15	CXCL10	GDF15
**AGE**	-0.06	**0.26**	0.16	**0.60**	0.12	0.11	-0.10	**0.53**
**GENDER**	**-0.30**	0.14	**-0.20**	0.18	**-0.40**	-0.03	-0.06	0.14
**BMI**	**-0.23**	0.11	-0.12	0.09	-0.18	0.05	**0.22**	0.03
**CK**	-0.14	-0.01	0.07	**0.22**	0.6	-0.08	**0.33**	-0.10
**CARDIO**	**-0.24**	**0.27**	-0.09	0.02	**-0.24**	**0.36**	**0.20**	-0.13
**GDF15**	0.02	x	0.04	x	-0.06	x	-0.07	x

CXCL10 and GDF15 levels are given in pg/ml serum, age in years. Correlations were scored negative (<0.2), weak (0.2 - 0.4) moderate (0.4 - 0.6). Correlations scored weak and above have been highlighted red when positive and green when negative. Body mass index (BMI), cardiovascular disease and hypertension (CARDIO), creatine kinase (CK), sporadic inclusion body myositis (IBM), immune mediated necrotizing myopathy (IMNM), hereditary muscle disorders (OTHER).

Table S4: Scoring myopathological changes in muscle biopsies from a selection of patients

ID	Muscle fiber necrosis		NNIF	Inflammation	
	severity	pattern	severity	severity	pattern
IMNM01	1	Advanced	0	0	
IMNM12	1		0	0	
IMNM13	2	Perifascicular, unevenly distributed	0	2	Perifascicular > perivascular
IMNM15	1		0	0	
IMNM16	1		1	1	
IMNM17	1		0	0	
IMNM18	1		0	0	
IMNM20	1		1	1	
IMNM21	2		1	2	Endomysial > perivascular
IBM01	1		1	1	Endomysial
IBM03	2		2	2	Endomysial
IBM04	1		2	1	Endomysial
IBM06	0		2	2	Endomysial
IBM07	1		2	2	Endomysial
IBM09	1		2	2	Endomysial
IBM14	2		2	2	Endomysial > perivascular
IBM15	1		2	2	Endomysial > perimysial > perivascular

Severity was scored absent (0), intermediate (1) to severe (2). Sporadic inclusion body myositis (IBM), immune mediated necrotizing myopathy (IMNM), nonnecrotic invaded muscle fibers (NNIF)
